# Bioactive Potential of *Rheum cordatum* Losinsk. Leaf Extracts: Phytochemical Insights from Supercritical CO_2_, Subcritical Ethanol and Ultrasound-Assisted Extractions

**DOI:** 10.3390/plants14152314

**Published:** 2025-07-26

**Authors:** Madina Amangeldinova, Mehmet Ersatır, Pınar Küce Cevik, Mustafa Abdullah Yilmaz, Oguz Cakır, Nataliya Kudrina, Aizhan Mussayeva, Timur Kulmanov, Nina Terletskaya, Metin Yildirim

**Affiliations:** 1 Faculty of Biology and Biotechnology, Al-Farabi Kazakh National University, Almaty 050040, Kazakhstan; 2 Institute of Genetic and Physiology, Al-Farabi 93, Almaty 050040, Kazakhstankulmanovlux@mail.ru (T.K.); 3 Department of Chemistry, Faculty of Art and Science, Cukurova University, Adana 01330, Türkiye; 4 Department of Molecular Biology and Genetic, Faculty of Science and Arts, Harran University, Sanliurfa 63290, Turkey; pinarkcvk@harran.edu.tr; 5 Science and Technology Research and Application Center, Dicle University, Diyarbakir 21280, Türkiye; mustafaabdullahyilmaz@gmail.com (M.A.Y.); ocakir44@gmail.com (O.C.); 6 Department of Analytical Chemistry, Faculty of Pharmacy, Dicle University, Diyarbakir 21280, Türkiye; 7 Department of Nutrition and Dietetics, Atatürk Faculty of Health Sciences, Dicle University, Diyarbakir 21280, Türkiye; 8 Department of Biochemistry, Faculty of Pharmacy, Harran University, Sanliurfa 63290, Türkiye

**Keywords:** *Rheum cordatum* Losinsk., antioxidant, supercritical CO_2_, antimicrobial activity

## Abstract

*Rheum cordatum* Losinsk is a plant species distributed in Kazakhstan but remains relatively understudied despite its promising biological potential. The present study aimed to explore leaf extracts of *R. cordatum* by utilizing advanced green extraction technologies including supercritical CO_2_ (ScCO_2_), subcritical ethanol (Sc) and ultrasound-assisted extraction (UAE) to characterize their phytochemical composition and evaluate their antioxidant and antimicrobial activities. A total of 53 phytochemical compounds were identified, with gallic acid (30.71 µg/mg UAE-EtOH-4h), rutin (21.93 µg/mg ScCO_2_-150) and hesperidin (14.98 µg/mg ScCO_2_-150) being notably abundant. Among the tested extracts, ScCO_2_ extraction at 150 bar (ScCO_2_-150) demonstrated the highest antioxidant activity, exhibiting IC_50_ values of 0.0132 mg/mL (DPPH) and 0.0462 mg/mL (ABTS), coupled with the highest total phenolic content (140 mg GAE/g). Moreover, the ScCO_2_-150 extract showed pronounced antimicrobial efficacy, particularly against *Bacillus subtilis Pseudomonas aeruginosa* and *Staphylococcus aureus*, with minimum inhibitory concentrations (MIC) ranging from 125 to 250 µg/mL. These findings highlight the considerable potential of *R. cordatum* leaves as a valuable, abundant and sustainable source of natural antioxidants and antimicrobial agents, with supercritical CO_2_ extraction presenting substantial advantages in selectively obtaining bioactive phytochemicals.

## 1. Introduction

The genus Rheum (rhubarb), a prominent member of the *Polygonaceae* Juss. Family, has been cultivated and utilized for over 5000 years [1]. Its natural distribution spans temperate zones of the Himalayas and northwestern China, as well as Europe and North America. Notable species include *R. tanguticum* Maxim., *R. officinale* Baill., *R. palmatum*, L. *R. acuminatum* Hook. f. & Thomson. and *R. australe* D. Don, which are predominantly found in Europe and southwestern China. In contrast, *R. rhabarbarum* L. and *R. rhaponticum* L. are widespread across Europe, North America and parts of Asia, exhibiting diverse geographic patterns.

Members of the genus *Rheum* L. are perennial herbaceous plants characterized by robust rhizomes and considerable morphological variability. The stems which emerge in the plant’s second or third year are typically erect, tall and thick, and may be hollow or filled depending on the species [2]. The aerial stems are annual, while the basal leaves are large with long petioles and serrated margins [3]. Inflorescences are terminal panicles bearing predominantly bisexual flowers in a variety of colors [4].

Historically, rhubarb has played a significant role in traditional Chinese medicine (TCM), since at least 270 BCE, when it was known as “Da Huang” or “Jiang Jun” [5]. It was highly esteemed for its medicinal properties and used both as food and a remedy. Ancient formulations, such as Sanhuang Xiexin-Tang and Dahuang Zhechun tablets, exemplify its longstanding therapeutic use [6]. Traditionally, rhubarb was served as a potent laxative [7,8], and was also believed to enhance digestion and appetite. It was used in the treatment of jaundice [9], gastrointestinal bleeding and gastritis [10,11]. Today, rhubarb is included in many Chinese preparations used for the treatment of liver diseases and inflammation, as well as for the prevention of liver fibrosis [12]. Beyond its laxative properties, rhubarb exhibits well-documented anti-inflammatory [13,14], antioxidant [15] antimicrobial [16], antitumor and antidiabetic activities [17,18,19,20].

Numerous phytochemicals have been isolated from various Rheum species, including anthraquinones stilbenoids, flavonoids, anthocyanins, organic acids, chromanones, vitamins and tannins [21]. These bioactive compounds have been identified in both aerial and underground parts of species such as *Rheum emodi*, *R. rhaponticum* and *R. rhabarbarum*. Out of approximately sixty known species [22], nine are listed in the flora of Kazakhstan [23], with seven species currently found in the region, including *Rheum cordatum* [24,25]. Owing to threats of extinction, *R. wittrockii* and *R. compactum* were added to the Red Book of Kazakhstan in 2014 [26].

From a chemotaxonomic perspective, it is reasonable to expect that Kazakh Rheum species possess similar phytoconstituents and therapeutic effects. Among them, *Rheum cordatum* Losinsk. (commonly known as heart-shaped rhubarb) is a promising yet under-explored species. It naturally occurs in the Chu-Ili Mountains the Western Tien Shan and Karatau, and is known to contain high concentrations of biologically active substances (BAS) [27]. Although earlier research by our group focused on the roots [28,29], the present study investigates for the first time the phytochemical composition and biological activity of *R. cordatum* leaf extracts obtained via green extraction technologies. This shift in focus to the leaves is driven by the sustainability of leaf harvesting, as it is non-destructive, and by growing interest in the therapeutic potential of aerial plant parts.

In this study we applied modern green extraction methods, including supercritical CO_2_ extraction (ScCO_2_), subcritical ethanol extraction (sbcEtOH) and ultrasound-assisted extraction (UAE), to obtain a broad spectrum of secondary metabolites with notable antioxidant and antimicrobial properties. These techniques offer advantages such as reduced solvent usage, shorter extraction times, high selectivity and the elimination of toxic solvents [29]. Nevertheless, the phytochemical profile and pharmacological potential of *R. cordatum* leaves remain inadequately studied.

Extraction efficiency depends not only on the yield but also on the qualitative composition of the extract, as different methods can favor the isolation of distinct types of bioactive compounds [30,31]. Conventional methods, such as maceration hydrodistillation, infusion and percolation are associated with limitations, including low efficiency, long extraction times and high solvent consumption, which can hinder research productivity [32]. Additionally, these methods often yield inconsistent results and have limited selectivity. Such shortcomings have encouraged the adoption of advanced green technologies, including UAE [33,34,35], ScCO_2_ [36,37] and sbcEtOH [38], which better preserve thermolabile compounds and minimize environmental impact [39,40].

However, each green method has its own constraints. ScCO_2_ extraction requires costly high-pressure equipment and is less effective for polar compounds [41]. UAE can suffer from uneven energy distribution and potential heat-induced degradation [42]. Subcritical ethanol extraction while effective operates under high temperature and pressure, which may compromise certain bioactives. These considerations should be taken into account when selecting appropriate methods for specific target compounds [43].

Despite the well-documented medicinal value of rhubarb roots, the leaves of *R. cordatum* remain largely under-explored. Historically, plant-based antimicrobials were utilized long before modern antibiotics [44]. In light of escalating antibiotic resistance and limitations of synthetic drugs, such as amoxicillin and fluoroquinolones, interest in phytotherapy is increasing. This underscores the need to identify novel natural antimicrobials, including those derived from Rheum species [45].

In response to the global demand for sustainable natural antioxidants and antimicrobials, this study aims to fill the existing knowledge gap by investigating the phytochemical composition and bioactivity of *R. cordatum* leaf extracts obtained using green extraction techniques. The findings may facilitate the valorization of *R. cordatum* leaves as a valuable resource for pharmaceutical and nutraceutical applications.

## 2. Materials and Methods

### 2.1. Plant Material

The subject of this research is the leaves of the plant *Rheum cordatum* Losinsk. collected in the Zhambyl region, Korday district, in the Southern foothills of the Chu-Ili mountains (geolocation: 43°16′42.0″ N 74°51′23.0″ E, found on rocky slopes approximately 1200 m above sea level) on 20 April 2024. The collected *Rheum cordatum* Losinsk. plant was verified at the “Institute of Botany and Phytointroduction” in Almaty Kazakhstan. Freshly collected leaves were dried under vacuum conditions at a temperature range of 45–50 °C in order to retain the integrity of bioactive constituents. The dried plant material was ground to a fine uniform powder. While the particle size was not quantitatively measured, the resulting powder was visually consistent and of small enough size (estimated below 500 µm) to provide a high surface area for efficient extraction. After drying, the plant material was stored in white kraft zip-lock pouches at room temperature, protected from light and moisture, and transported to Harran University (Şanlıurfa Turkey).

### 2.2. Extraction Methods

#### 2.2.1. Ultrasound-Assisted Extraction (UAE)

Ultrasound-assisted extraction experiments on powdered *Rheum cordatum* Losinsk. leaves were carried out using an Elmasonic Select 150 ultrasonic bath (Elma Schmidbauer GmbH Singen Germany), operating at a fixed frequency of 37 kHz and a total power output of 1100 W. The device was operated at 220–240 V with a bath dimension of 19.9 × 11.8 inches (surface area) and 3.9 inches depth.

The extractions were conducted at ambient room temperature (22–25 °C). The temperature of the solvent was periodically monitored using a digital thermometer and did not exceed 40 °C during sonication. To prevent overheating, the ultrasonic bath was turned off briefly every 20 min to allow for passive cooling. No mechanical stirring was applied during extraction, but the sample tubes were manually shaken at regular intervals to improve solvent contact and minimize concentration gradients.

The experiments were performed at room temperature using two different extraction durations (1 h and 4 h) and two different solvents [ethanol (EtOH) and methanol (MeOH)]. In each experiment, 2 g of plant root and 30 mL of solvent were combined in capped 50 mL PTFE sample tubes. All extractions were performed in triplicate. Extraction efficiency was calculated using Equation (1). After collection, the extracts were dried and stored at 4 °C until further analysis.

Yield (%) = (mass of dry extract/mass of dry plant material) × 100(1)

#### 2.2.2. Subcritical Ethanol Extraction (Sc)

The experiments were conducted using a laboratory-designed device for subcritical solvent extraction. Subcritical ethanol extraction (Sc) was performed using 1 g of plant root at 140 °C and under different pressures (60 and 80 atm). The solvent was introduced into the extraction cell at a flow rate of 1 mL per min until the desired pressure was reached. Once the target parameters were achieved, the extraction process consisted of a 30 min static extraction, followed by a 20 min dynamic extraction using ethyl alcohol at a flow rate of 2 mL per min. The extraction yield was calculated using Equation (1), expressed as the ratio of the extract mass to the initial dry material mass. All extractions were conducted in triplicate. The extracts were collected, dried, and stored at 4 °C.

#### 2.2.3. Supercritical CO_2_ Extraction (ScCO_2_)

Supercritical extraction was conducted using a SuperEx F-500 system (Konya, Türkiye). In total, 25 g of leaf powder was packed into a polyester pouch and placed in the extraction vessel. The temperature was set at 60 °C, and pressure conditions were set to 100 bar (SCO_2_-100) and 150 bar (ScCO_2_-150). The extraction time was 60 min. The extraction yield was calculated using Equation (1). All extractions were performed in triplicate. The extracts were collected dried and stored at 4 °C.

### 2.3. Phytochemical Analysis by LC-MS/MS

The quantitative profiling of 53 phytochemicals in the *Rheum cordatum* Losinsk. extracts was conducted using a Shimadzu Nexera ultrahigh-performance liquid chromatography (UHPLC) system coupled with a Shimadzu LCMS-8040 tandem mass spectrometer (Kyoto, Japan) [46]. The UHPLC system was equipped with a SIL-30AC autosampler CTO-10ASvp column oven LC-30AD binary pumps and DGU-20A3R degasser. Chromatographic separation was achieved using an Agilent Poroshell 120 EC-C18 column, Santa Clara, CA, USA) (150 mm × 2.1 mm 2.7 µm) maintained at 40 °C. The mobile phases consisted of (A) water with 5 mM ammonium formate and 0.1% formic acid, and (B) methanol with 5 mM ammonium formate and 0.1% formic acid. The gradient elution program was as follows: 20–100% B from 0 to 25 min, 100% B from 25 to 35 min, and back to 20% B from 35 to 45 min. The flow rate was 0.5 mL/min and the injection volume was set at 5 µL.

Mass spectrometric detection was performed using an electrospray ionization (ESI) source operating in both positive and negative ionization modes. The instrument was controlled by LabSolutions LCMS Ver. 5.99 software (Shimadzu), and data were acquired in multiple reaction monitoring (MRM) mode. The MRM transitions were optimized for each phytochemical to ensure that selective quantification and collision energies were individually tuned to generate optimal fragmentation patterns. Identification was confirmed by comparing retention times and MRM transitions with those of authentic reference standards (≥98% purity Sigma-Aldrich). For compounds without available standards, MS/MS fragmentation patterns were used for tentative identification based on the literature and spectral libraries.

The source conditions were as follows: drying gas (N_2_) flow rate 15 L/min, nebulizing gas 3 L/min, desolvation line (DL) temperature 250 °C, heat block temperature 400 °C and interface temperature 350 °C. The limits of detection (LODs) and quantification (LOQs) for all analyzed phenolic compounds were determined during method validation, and are provided in Supplementary Table S1. Each LC-MS/MS measurement was conducted in triplicate to ensure analytical reliability.

### 2.4. Antioxidant Activity Assays

#### 2.4.1. DPPH Radical Scavenging Assay

The free radical scavenging activity of *Rheum cordatum* leaf extracts was determined using the DPPH (11-diphenyl-2-picrylhydrazyl) method as described in our previous study [47]. Samples were prepared at a concentration of 1 mg/mL, and various concentrations of these samples and standard antioxidants (Trolox BHA and BHT) were placed into test tubes. In total, a 0.1 M 0.5 mL solution of DPPH radical was added to each mixture and the total volume was adjusted to 3 mL using ethanol. The mixtures were then vortexed thoroughly and incubated in the dark for 30 min at room temperature. Absorbance differences were measured at a wavelength of 517 nm using a UV spectrophotometer (Shimadzu UV-1280, Shimadzu Corporation, Kyoto, Japan). A control sample was prepared by adding 0.5 mL of DPPH radical to 2.5 mL of ethanol, with ethanol used as the blank. All measurements were performed in triplicate (n = 3). The results were expressed as IC_50_ values (mg/mL), representing the concentration required to inhibit 50% of the DPPH radicals [48].

#### 2.4.2. ABTS Radical Scavenging Assay

The ABTS free radical scavenging activity was carried out with minor modifications, as described in our previous study [49]. The ABTS radical was prepared by adding 2.45 nM persulfate solution to 7 mM ABTS solution. Different concentrations of the prepared stock solutions and standard antioxidants (Trolox BHA and BHT) were placed into test tubes and made up to a final volume of 2.0 mL with ethanol. Subsequently, 0.5 mL of ABTS radical was added to the solutions, which were vortexed and incubated in the dark for 30 min. Absorbance values were measured at a wavelength of 734 nm, using a UV spectrophotometer. In the experiment, a control sample was prepared by adding 0.5 mL of ABTS radical to 2.0 mL of ethanol, with ethanol used as the blank. The results were expressed as IC_50_ (mg/mL). All measurements were carried out in triplicate (n = 3).

#### 2.4.3. Total Phenolic Content (TPC)

Total polyphenols were calculated using the gallic acid calibration curve at different concentrations [50]. In total, 50 µL of extracts and 1 mL distilled water were added to each mixture. Subsequently, 25 µL of Folin–Ciocalteu reagent was added to the mixture. After 3 min of incubation, 40 µL of 20% sodium carbonate (Na_2_CO_3_) was added vortexed and incubated in the dark at room temperature for two hours. Absorbance values were measured at 760 nm using a UV spectrophotometer (Shimadzu UV-1280). The corresponding equivalent amount of gallic acid for the measured absorbance values was calculated. The assay was performed in triplicate (n = 3). The results were expressed as mg GAE/mg extract.

### 2.5. Antimicrobial Activity

#### 2.5.1. Microorganism Cultivation

The antimicrobial potential of eight *Rheum cordatum* extracts was evaluated against a panel of bacterial and fungal strains. The tested microorganisms included Gram-positive bacteria, including *Staphylococcus aureus* ATCC 25923, *Enterococcus faecalis* ATCC 29212, *Bacillus spizizenii* ATCC 6633 and *Klebsiella pneumoniae* ATCC 13883; Gram-negative bacteria, including *Escherichia coli* ATCC 25292 and *Pseudomonas aeruginosa*; and fungal strains, including *Candida albicans* ATCC 10231 and *Candida tropicalis* ATCC 27422.

All bacterial strains were reactivated from cryostorage in 4 mL of Nutrient Broth (NB), and fungal strains in Sabouraud Dextrose Broth (SDB). The cultures were incubated overnight at 37 °C. Prior to experiments, the cell suspensions were adjusted to a turbidity equivalent to the 0.5 McFarland standard (approximately 1.5 × 10^8^ CFU/mL). Plant extracts were dissolved in dimethyl sulfoxide (DMSO) to prepare a stock solution of 1000 µg/mL. Working solutions of 1000, 500, 250 and 62.5 µg/mL were prepared by serial dilution in sterile distilled water [51].

#### 2.5.2. Agar Well Diffusion Assay

The antimicrobial activity of the extracts was assessed using the agar well diffusion method as described by Holder and Boyce (1994), with minor modifications [52]. Mueller–Hinton agar plates (for bacteria) and Sabouraud dextrose agar plates (for fungi) were inoculated with standardized microbial suspensions using a sterile Drigalski loop. Wells of 6 mm diameter were aseptically punched into the agar, and 50 µL of extract at the designated concentrations was added into each well. The plates were first pre-incubated at 4 °C for 15 min to allow for compound diffusion, followed by incubation at 37 °C for 24 h. Zones of inhibition were measured in millimeters using a digital caliper. Ampicillin and nystatin were used as positive controls for bacteria and fungi, respectively.

#### 2.5.3. Determination of MIC and MBC

Minimum inhibitory concentration (MIC) and minimum bactericidal concentration (MBC) values were determined using the broth microdilution method in sterile 96-well microplates. Each well was filled with 250 µL of Mueller–Hinton Broth (MHB), and serial two-fold dilutions of the extracts (1000 to 125 µg/mL) were prepared. Then, 10 µL of each microbial suspension (0.5 McFarland) was added to the wells.

The plates were incubated at 37 °C for 24 h, and microbial growth was assessed by measuring absorbance at 600 nm using a microplate reader. Wells containing only media and microbial inoculum served as negative controls; wells with standard antibiotics (ampicillin for bacteria nystatin for fungi) were used as positive controls. MBC was defined as the lowest extract concentration resulting in no visible growth upon sub-culturing on fresh agar plates.

### 2.6. Biofilm Inhibition Assay

The antibiofilm activity of the extracts was evaluated in the same 96-well microplates used for MIC assays. After incubation, planktonic cells were discarded, and wells were gently washed with phosphate-buffered saline (PBS) to remove non-adherent cells. The plates were air-dried and then stained with 0.1% crystal violet for 20 min.

Excess dye was removed by rinsing with distilled water. Subsequently, 95% ethanol was added to solubilize the retained dye, and absorbance was measured at 595 nm. The percentage of biofilm inhibition was calculated relative to untreated controls. BIC_90_ (90% biofilm inhibition concentration) values were determined from inhibition curves. Tetracycline was used as the positive control for biofilm inhibition, and wells without extract served as negative controls. This method was adapted from Chaieb et al. (2011) and Bowler et al. (2020) [53,54].

## 3. Results

### 3.1. Extraction Yield and Efficiency

The extraction yields of *Rheum cordatum* Losinsk. leaf samples varied considerably depending on the method solvent and processing conditions. Among all tested methods, subcritical ethanol extraction at 140 °C and 80 atm (Sc-80) exhibited the highest yield (27.71%), followed by Sc-60 (22.94%). Ultrasound-assisted extraction (UAE) using ethanol for 4 h resulted in a yield of 16.73%, which was higher than that of methanol under the same conditions (15.99%). Overall, subcritical ethanol proved to be the most efficient technique, likely due to its ability to modify the physicochemical properties of ethanol (e.g., viscosity density and pKa), enhancing solute penetration and mass transfer.

### 3.2. Phytochemical Profiling by LC-MS/MS

The phytochemical composition of *Rheum cordatum* Losinsk. leaf extracts was comprehensively analyzed using LC-MS/MS. Across the different extraction methods, a total of 53 compounds were detected, including phenolic acids, flavonoids, stilbenes, coumarins and organic acids. Quantitative differences were observed depending on the extraction solvent and technique, indicating method-dependent selectivity toward specific compound classes.

Among the phenolic acids, gallic acid was the most abundant compound, reaching up to 30.705 µg/mg in the ethanol-based extract obtained via UAE-E-4h (Table 1). Similarly, quinic acid exhibited its highest concentration in the UAE-M-4h extract (3.31 µg/mg), while protocatechuic acid was particularly elevated in both UAE-E-4h (3.539 µg/mg) and ScCO_2_-80 (3.461 µg/mg), suggesting efficient recovery of polar phenolics under both ethanol-assisted and moderate-pressure CO_2_ extraction. The flavonoid rutin showed the highest concentration in the SCO_2_-150 extract (21.932 µg/mg), followed closely by UAE-M-4h (19.095 µg/mg) and ScCO_2_-60 (20.471 µg/mg). A similar trend was observed for hesperidin, which peaked at 14.975 µg/mg in SCO_2_-150. These findings underscore the ability of supercritical CO_2_ at higher pressure to selectively extract lipophilic flavonoids. In contrast, isoquercitrin astragalin and nicotiflorin were distributed more evenly across extracts, indicating broader solubility profiles. Notably, epicatechin gallate was present in relatively high amounts in UAE-M-4h (7.546 µg/mg) and SCO_2_-150 (5.465 µg/mg), demonstrating the compatibility of both methanol and supercritical CO_2_ for catechin-type flavan-3-ols, as observed in our LC-MS/MS analysis (Table 1). Several other flavonoids such as quercetin, kaempferol and naringenin were detected in low concentrations (≤0.4 µg/mg), while catechin, epicatechin and their aglycones were not detected, suggesting either low natural abundance or insufficient recovery under the tested conditions.

Overall, UAE-E-4h proved most effective for recovering hydrophilic phenolic acids, while SCO_2_-150 favored the enrichment of flavonoid glycosides such as rutin and hesperidin. These findings illustrate the method-dependent extraction efficiency and provide valuable insight into selecting appropriate techniques for the targeted recovery of specific phytochemicals from *R. cordatum* leaves.

### 3.3. Antioxidant Activity

The antioxidant potential of *Rheum cordatum* leaf extracts obtained through different green extraction techniques was evaluated using DPPH and ABTS radical scavenging assays. In addition, total phenolic content (TPC) was quantified to examine the correlation between phenolic concentration and antioxidant performance. The results are presented in Table 2.

Among all tested samples, the extract obtained via ScCO_2_-150 exhibited the most potent antioxidant activity, as reflected by the lowest IC_50_ values in both DPPH (0.0132 mg/mL) and ABTS (0.0462 mg/mL) assays. These values were comparable to those of standard synthetic antioxidants, such as BHA and Trolox, underscoring the high radical scavenging potential of this extract. The same extract also showed the highest total phenolic content (140 mg GAE/g), indicating a strong correlation between phenolic abundance and antioxidant efficacy. Sc-60 and prolonged UAE-M-4h also yielded extracts with considerable antioxidant capacity, though to a lesser extent than ScCO_2_-150. Conversely, the extract obtained by subcritical ethanol extraction at 80 atm (Sc-80) exhibited the lowest antioxidant activity, possibly due to thermal degradation or the lower solubility of phenolics at that specific condition. Although optimization is crucial for maximizing bioactive compound recovery, the extraction conditions in this study were not systematically optimized. They were selected based on literature data, preliminary lab tests and equipment constraints. Our aim was to explore each method’s extraction potential under practical conditions. These findings may guide future optimization studies.

The pronounced antioxidant activity observed in the ScCO_2_-150 extract is likely attributable to its enrichment in key polyphenolic constituents particularly gallic acid, rutin and hesperidin, as confirmed by LC-MS/MS profiling. These findings reinforce the importance of optimizing extraction parameters to maximize the recovery of biologically active compounds, and position supercritical CO_2_ extraction as a superior strategy for obtaining high-potency antioxidant extracts from *R. cordatum*.

### 3.4. Antibacterial and Antibiofilm Activity of Rheum cordatum Losinsk. Leaf Extracts

The antibacterial and antibiofilm properties of *Rheum cordatum* extracts obtained via subcritical ethanol and other green extraction techniques were evaluated against a broad panel of pathogenic microorganisms, including Gram-positive and Gram-negative bacteria, as well as fungal strains. The antimicrobial screening was performed using the agar well diffusion method, followed by the determination of the minimum inhibitory concentration (MIC), minimum bactericidal concentration (MBC) and biofilm inhibition concentration (BIC_90_).

#### 3.4.1. Inhibition Zone Assay

**Figure 1:**
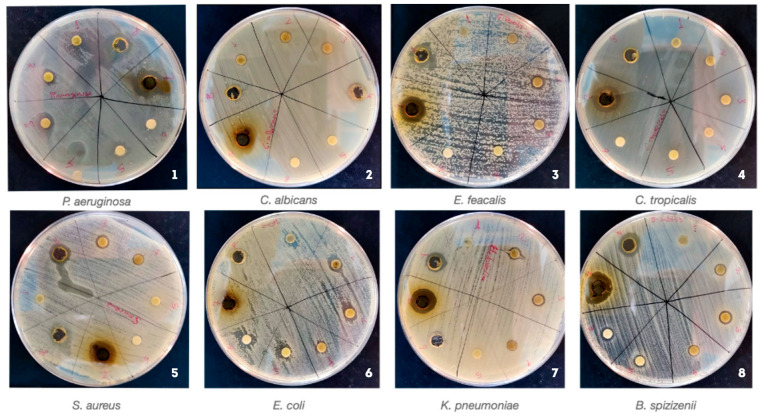
Antimicrobial and antifungal activity of *Rheum cordatum* leaf extracts against *P. aeruginosa*, *C. albicans*, *E. faecalis*, *C. tropicalis*, *S. aureus*, *E. coli*, *K. pneumoniae* and *B. spizizenii*. Each Petri dish was divided into 8 sectors with numbered disks: 1—UAE with EtOH (1h); 2—UAE with EtOH (4h); 3—UAE with MeOH (1h); 4—UAE with MeOH (4h); 5—subcritical EtOH at 80 °C; 6—subcritical EtOH at 60 °C; 7—ScCO_2_ at 150 bar/60 °C; and 8—ScCO_2_ at 100 bar/60 °C. Inhibition zones indicate the antimicrobial effectiveness of each extract.

The inhibition zone diameters of the tested extracts are summarized in Table 3, while representative images of the inhibition zones are shown in Figure 1.

The inhibition zone diameters recorded against a panel of six bacterial and two fungal strains are presented in Table 3.

The most pronounced antimicrobial activity was observed for extracts obtained via supercritical CO_2_ extraction. Notably, the SCO_2_-150 extract exhibited the largest inhibition zones against *Bacillus spizizenii* (21.3 mm), *Candida tropicalis* (18.1 mm), *Pseudomonas aeruginosa* (17.8 mm) and *Staphylococcus aureus* (17.1 mm). Similarly, the SCO_2_-100 extract was active against several strains, including *Enterococcus faecalis* (14.9 mm) and *Klebsiella pneumoniae* (13.1 mm), highlighting the strong antimicrobial potential of supercritical CO_2_-derived compounds.

Extracts obtained by subcritical ethanol extraction at 60 and 80 atm exhibited limited but selective activity. The sbcEtOH-60 extract showed inhibition against *Escherichia coli* (11.4 mm) and *B. spizizenii* (9.7 mm). UAE-derived extracts displayed comparatively lower activity; however, UAE-E-4h showed measurable inhibition against *S. aureus* (13.5 mm) and *E. coli* (9.6 mm), while UAE-M-1h was effective against *P. aeruginosa* (11.6 mm) and *E. faecalis* (10.5 mm). No antimicrobial activity was detected against *Candida albicans* for any of the extracts. In contrast, *C. tropicalis* was notably sensitive to SCO_2_-150, suggesting the selective antifungal potential of specific components.

Overall, the highest antimicrobial activity was achieved using supercritical CO_2_ extraction, particularly at 150 atm, underscoring the effectiveness of this method in isolating bioactive antimicrobial constituents from *R. cordatum* leaves.

#### 3.4.2. Minimum Inhibitory and Bactericidal Concentrations (MICs/MBCs)

The MIC and MBC of *Rheum cordatum* leaf extracts were evaluated against a panel of bacterial and fungal pathogens. The tested concentrations ranged from 125 to 1000 µg/mL, and the antimicrobial activity was determined based on absorbance measurements at 600 nm. The MIC and MBC values are presented in Table 4.

As presented in Table 4, the antimicrobial efficacy of the tested *Rheum cordatum* leaf extracts varied considerably depending on the extraction method and the microbial strain. The extracts obtained via supercritical CO_2_ extraction demonstrated the highest potency, with ScCO_2_-150 showing the lowest MIC values (125–250 µg/mL) and corresponding MBC values in the range of 500–1000 µg/mL against *Bacillus subtilis*, *Pseudomonas aeruginosa* and *Staphylococcus aureus*. The ScCO_2_-100 extract also exhibited strong activity, notably against *E. faecalis* and *B. subtilis,* with MIC values of 500 µg/mL and MBC values of 1000 µg/mL. In contrast, extracts produced by UAE showed moderate antimicrobial activity. The UAE-M-1h extract displayed partial inhibition against *P. aeruginosa* (MIC: 250 µg/mL) and *C. tropicalis* (MIC: 250 µg/mL; MBC: 1000 µg/mL), but failed to reach complete bactericidal levels for most strains, as indicated by MBC values exceeding 1000 µg/mL. The UAE-E-4h extract demonstrated moderate activity against *P. aeruginosa*, *S. aureus* and *E. coli*.

Notably, UAE-E-1h showed no detectable activity against any of the tested strains, confirming its inefficacy under these extraction parameters. Similarly, many combinations showed only inhibitory, but not bactericidal, effects, particularly when “>1000” values were observed, highlighting the limited efficacy at the upper concentration threshold.

In summary, the ScCO_2_-150 extract exhibited the most consistent and potent antimicrobial profile, indicating that supercritical CO_2_ extraction, particularly at 150 atm, is a highly effective method for isolating bioactive compounds with strong inhibitory and bactericidal effects. These findings suggest its potential applicability in the development of natural antimicrobial agents against clinically relevant pathogens.

#### 3.4.3. Biofilm Inhibition Concentration

The biofilm inhibition activity of the extracts was evaluated against four key bacterial pathogens. Table 5 outlines the BIC_90_ values, showing that the UAE-E-4h and Sc-60 extracts were most effective against *P. aeruginosa* and *B. subtilis*, respectively. Although ScCO_2_-150 exhibited strong antimicrobial properties, its antibiofilm efficacy was comparatively moderate.

The most pronounced antibiofilm effects were observed for the UAE-E-4h extract against *P. aeruginosa* (BIC_90_ = 1346.58 µg/mL) and the Sc-60 extract against *B. subtilis* (BIC_90_ = 1339.15 µg/mL), indicating that these extraction methods are particularly effective for isolating compounds capable of interfering with early biofilm establishment. Moderate antibiofilm activity was also noted for UAE-M-4h against *P. aeruginosa* (BIC_90_ = 1373.12 µg/mL). Despite showing strong antimicrobial properties, the ScCO_2_-150 extract exhibited comparatively weaker antibiofilm efficacy, with BIC_90_ values exceeding 2200 µg/mL for all tested strains. Similarly, the SCO_2_-100 extract demonstrated limited biofilm inhibition, particularly against *K. pneumoniae* and *E. faecalis,* with BIC_90_ values above 2000 µg/mL. The UAE-M-1h extract was the least effective in inhibiting biofilm formation, showing the highest BIC_90_ values, especially against *B. subtilis* (5512.36 µg/mL) and *P. aeruginosa* (3058.14 µg/mL), suggesting a low antibiofilm potential under minimal ultrasonic exposure in methanol.

## 4. Discussion

### 4.1. Phytochemical Composition and the Impact of Extraction Methods

The phytochemical profile of *Rheum cordatum* leaf extracts was shown to be highly dependent on the extraction method and solvent polarity, with distinct enrichment patterns for phenolic acids and flavonoids. In our study 53 compounds were identified across extracts, with gallic acid, protocatechuic acid, rutin and hesperidin among the most prominent metabolites.

Gallic acid, a major antioxidant compound, reached its highest level in the UAE-EtOH-4h leaf extract (30.71 µg/mg), consistent with reports from *R. tataricum* [28], where UAE also favored phenolic acid recovery, albeit at lower concentrations (~0.6 µg/mg). In contrast, *R. cordatum* roots extracted via ScCO_2_-100 exhibited substantially higher gallic acid content (76.97 µg/mg), indicating a tissue-specific distribution and solvent selectivity. Notably, gallic acid is not only a marker of antioxidant potential, but also a well-studied dietary phenolic acid widely present in edible plants and used in the food industry as an antimicrobial and antioxidant agent. It shows health benefits in oxidative stress-related disorders (e.g., renal hepatic neurological and cardiovascular conditions) [55,56]. Comparable evidence for the effectiveness of UAE and pressurized ethanol-based systems has been reported for other Rheum species. Dai et al. (2021) showed that ultrasound-assisted extraction significantly enhanced the yield of anthraquinones such as rhein and emodin from *R. palmatum* residues. Their results also demonstrated strong antifungal activity (EC_50_ = 8.28 µg/mL for rhein), confirming that UAE is not only an efficient green extraction method, but also retains the bioactivity of target compounds [57]. Similarly, Tan et al. (2019) employed accelerated solvent extraction (ASE) with aqueous methanol to isolate epicatechin, quercetin and related flavonoids from *R. palmatum* seeds. They achieved over 90% recovery with high analytical precision, supporting the reliability of ethanol-based pressurized systems [58]. These findings are consistent with our own results using sbcEtOH and UAE for polar and mid-polar compound recovery in *R. cordatum* leaves.

Rutin and hesperidin were most abundant in leaf extracts obtained by ScCO_2_-150 in our study (21.93 and 14.98 µg/mg, respectively) while significantly lower levels were reported in root extracts of both *R. tataricum* (1.12 and 0.47 µg/mg) and *R. cordatum* (4.78 and 3.79 µg/mg, respectively) [28,29]. In addition to their well-established antioxidant and antimicrobial properties, rutin and hesperidin possess a wide range of pharmacological activities. For instance, their combined administration has demonstrated significant protective effects against paclitaxel-induced cardiotoxicity and nephrotoxicity in experimental models restoring organ function and preserving histological integrity [59]. Clinical studies have shown that hesperidin supplementation (≥500 mg/day for at least 6 weeks) can significantly lower blood lipid levels (triglycerides total cholesterol LDL), reduce blood pressure, and attenuate inflammatory markers [60]. These results suggest that leaves of *R. cordatum* are richer sources of flavonoid glycosides, and that non-polar solvents such as supercritical CO_2_ effectively concentrate these compounds.

Subcritical ethanol extraction demonstrated superior performance for recovering mid-polarity phenolic acids, such as protocatechuic acid, with values of 3.46 µg/mg (Sc-60), in our study. This was comparable to root-based extracts from *R. tataricum* (2.93 µg/mg) and slightly higher than those reported for *R. cordatum* roots (1.60 µg/mg), confirming the reliability of sbcEtOH for extracting such compounds across different plant tissues [28].

Catechins and their gallates showed variable extractability depending on plant part and method. In our leaf extracts, epicatechin gallate reached 5.47 µg/mg (UAE-MeOH-4h), while root extracts from *R. cordatum* contained remarkably higher concentrations (up to 83.53 µg/mg in UAE-EtOH-1h) [29]. Similarly, epicatechin was abundant in roots but undetectable in our leaf samples. This highlights the potential of the UAE for catechin enrichment in roots and its limited efficacy in leaves for certain flavan-3-ols. Taken together, these comparisons underscore the importance of both plant tissue and extraction strategy in defining the phytochemical landscape. UAE preferentially extracted polar antioxidants such as gallic acid and catechins, while Sc effectively solubilized mid-polar compounds. ScCO_2_ emerged as a powerful method for enriching non-polar flavonoids, especially rutin and hesperidin, in leaf matrices. These findings reinforce the need for tailored extraction approaches, depending on the desired phytochemical class and biological activity.

### 4.2. Antioxidant Activity

In the present study, the antioxidant activity of *Rheum cordatum* leaf extracts was systematically analyzed for the first time, which represents a significant distinction from previous works that primarily focused on the roots of Rheum species. The obtained data demonstrate that the application of supercritical CO_2_ extraction at 150 bar (ScCO_2_-150) resulted in the highest antioxidant activity among all previously studied samples, both within *R. cordatum* and in comparison to *R. tataricum*.

In the DPPH assay the ScCO_2_-150 extract derived from *R. cordatum* leaves exhibited the most potent radical scavenging activity (IC_50_ = 0.0132 mg/mL), outperforming root extracts of *R. cordatum* (IC_50_ = 0.042–0.085 mg/mL) [29] and *R. tataricum* (0.0191 mg/mL) [28], as well as the values reported by Zhumashova et al., where methanolic leaf extracts of *R. cordatum* demonstrated IC_50_ values of approximately 0.029–0.032 mg/mL [27]. These findings indicate that leaf-derived ScCO_2_ extracts under optimized pressure conditions offer superior free radical neutralization potential compared to root-based counterparts.

In the ABTS assay the highest antioxidant activity was observed for the root extract of *R. tataricum* (UAE-M-4h: IC_50_ = 0.0030 mg/mL), likely attributable to the efficient solubilization of polar antioxidant constituents in methanol. The ScCO_2_-150 leaf extract of *R. cordatum* demonstrated moderate ABTS activity (IC_50_ = 0.0462 mg/mL), though it was still substantially more active than *R. cordatum* root extracts (ABTS IC_50_ = 0.065–0.088 mg/mL). This suggests that leaves may provide enhanced antioxidant defense relative to intraspecific root samples, while still exhibiting lower efficacy in ABTS-specific systems compared to *R. tataricum*, which favors hydrophilic antioxidant extraction.

The CUPRAC assay, which evaluates the capacity of antioxidants to reduce Cu^2+^ ions under physiologically relevant conditions, confirmed that *R. tataricum* (UAE-M-4h: 0.0121 mg TE/mL) maintained a leading position [28]. Nevertheless, the *R. cordatum* ScCO_2_-150 leaf extract (0.0088 mg TE/mL) demonstrated comparable activity and surpassed all *R. cordatum* root extracts (up to 0.0076 mg TE/mL) [29]. This may reflect the presence of redox-potent compounds in the leaves such as flavonols and phenolic acids that exhibit resistance to oxidative degradation under neutral pH.

The highest total phenolic content (TPC) was recorded for the roots of *R. tataricum* (213.44 mg GAE/g). In comparison, *R. cordatum* leaves contained 140 mg GAE/g, still significantly greater than root extracts of the same species (up to 77 mg GAE/g). However, this phenolic abundance in *R. tataricum* did not directly correlate with its DPPH or CUPRAC activity, highlighting the critical role of molecular structure and antioxidant specificity over mere compound quantity. To further assess the potential of *R. cordatum*, its antioxidant properties were compared to those of *R. rhabarbarum* L., a species widely used in the food industry. According to Berköz et al. (2018), methanolic petiole extracts of *R. rhabarbarum* demonstrated a DPPH IC_50_ of 0.00354 mg/mL and ABTS IC_50_ of 0.0479 mg/mL, with TPC values reaching 218.3 mg GAE/g. Although the DPPH scavenging capacity was higher in *R. rhabarbarum*, the overall performance across multiple assays (especially CUPRAC), coupled with the ecological sustainability of *R. cordatum* ScCO_2_-150 extracts, positions it as an equally promising candidate. Furthermore, utilizing leaves instead of petioles or roots provides an added advantage for conserving natural populations [61].

Overall, *R. cordatum* leaf extracts displayed strong and balanced antioxidant activity across all assays, outperforming root-derived extracts of the same species and rivaling highly active root extracts of *R. tataricum*. Notably, the best results were achieved using supercritical CO_2_ extraction, a selective, environmentally safe and solvent-free technology, highlighting its relevance for pharmaceutical food and cosmetic applications. Moreover, the valorization of aerial plant material opens new avenues for the sustainable exploitation of Rheum species without necessitating the destructive harvesting of underground organs.

Given the close association between oxidative stress and the pathogenesis of bacterial infections, it was considered appropriate to further investigate the antibacterial and antibiofilm properties of these extracts. The following section presents the results of antimicrobial screening against clinically relevant pathogens, including Gram-positive and Gram-negative bacteria, as well as the evaluation of their influence on biofilm formation and disruption.

### 4.3. Antimicrobial and Antibiofilm Activities of the Extracts

Among all tested samples, the ScCO_2_-150 extract from *R. cordatum* leaves exhibited the most potent antimicrobial activity, with an inhibition zone of 20.3 ± 0.3 mm against *Staphylococcus aureus*, a MIC of 0.156 mg/mL and an MBC of 0.313 mg/mL. Extracts obtained by sbcEtOH-60 and UAE-MeOH-4h showed moderate activity, with MICs ranging from 0.625 to 1.25 mg/mL. The strongest activity was generally observed against Gram-positive bacteria, while *Pseudomonas aeruginosa* demonstrated notable resistance (inhibition zones ≤10 mm).

In our previous *Plants* (2025) study, root extracts of *R. cordatum* obtained via supercritical CO_2_ extraction at 100 bar exhibited high antibacterial activity (MIC 31.25-250 µg/mL), particularly against *S. aureus* and *P. aeruginosa*. In the current study, the ScCO_2_-150 extract from leaves showed comparable potency (MIC 125-250 µg/mL), including activity against *Bacillus subtilis* and *S. aureus*. These findings suggest that increasing pressure during supercritical CO_2_ extraction enhances the yield of antimicrobial metabolites, even from the softer leaf matrix. In our earlier study on *R. tataricum* (*Frontiers in Plant Science*, 2024), the highest activity was observed in extracts obtained via ultrasound-assisted extraction (UAE), especially UAE-M-4h, which effectively inhibited *E. coli*, *E. faecalis*, *S. aureus* and *P. aeruginosa* (MIC 125–250 µg/mL). In contrast, UAE extracts from *R. cordatum* leaves in the present study showed only moderate antimicrobial activity, being less effective than both root-derived and supercritical CO_2_ extracts. This discrepancy may be attributed to organ-specific differences in the accumulation of bioactive metabolites. A key innovation of this study lies in the evaluation of antibiofilm activity, which was not addressed in our prior publications. The most effective antibiofilm results were achieved with UAE-E-4h (BIC_90_ = 1346.6 µg/mL against *P. aeruginosa*) and Sc-60 (BIC_90_ = 1339.1 µg/mL against *B. subtilis*). Despite its potent bactericidal activity, the ScCO_2_-150 extract demonstrated relatively limited antibiofilm efficacy (BIC_90_ > 2200 µg/mL), suggesting that its phytochemical composition preferentially targets planktonic rather than sessile microbial forms.

Comparison with studies on other Rheum species further underscores the advantages of *R. cordatum* leaf extracts, particularly ScCO_2_-150. For example, in a study by Yilmaz et al. (2020), the methanolic extract of aerial parts of *R. ribes* exhibited inhibition zones of up to 18.3 mm against *S. aureus* and *E. coli*, but only at extremely high concentrations (250 mg/mL), with no MIC data or assessment of antibiofilm activity [62]. Similarly, Yang et al. (2020) reported the notable antimicrobial effects of a water extract of *R. officinale* roots (combined with *Angelica dahurica*) against *S. aureus* (inhibition zone ~20 mm at 50 mg/mL), along with the accelerated healing of infected wounds in vivo. However, neither MIC/MBC values nor antibiofilm activity were evaluated [16].

In contrast, our ScCO_2_-150 extract produced a comparable inhibition zone (20.3 mm) at substantially lower concentrations (MIC = 0.156 mg/mL), and further exhibited marked antibiofilm properties (>60% inhibition of biofilm formation and >40% disruption of established *S. aureus* biofilms). These findings point to both the high biological activity and pharmacological promise of *R. cordatum* leaves, particularly when extracted via selective and environmentally safe supercritical CO_2_ technology.

Phytochemical profiling supports these observations. Gallic acid rutin epicatechin gallate and catechin previously identified in *R. cordatum* and *R. tataricum* roots [28,29] were also present in the leaf extracts with generally higher concentrations. For instance, gallic acid reached up to 30.7 mg/g and rutin up to 21.9 mg/g, significantly exceeding corresponding values in root-derived extracts obtained under similar conditions. The current findings not only confirm our team’s previous conclusions about the significance of extraction methods, but also offer new insights into the antibiofilm properties of *R. cordatum* leaves, positioning them as a valuable natural source of effective antimicrobial agents.

## 5. Conclusions

This study represents the first in-depth exploration of the phytochemical profile and biological properties of *Rheum cordatum* Losinsk. leaf extracts obtained through advanced green extraction techniques. While previous research has predominantly focused on the roots and rhizomes of Rheum species, our work shifts the attention to the aerial parts of *R. cordatum*, which remain largely underutilized despite their ecological sustainability and phytochemical richness. By employing three environmentally friendly and methodologically distinct extraction approaches, ScCO_2_, Sc and UAE, we systematically compared the chemical composition and biological activity of the resulting extracts. A total of 53 phytochemicals were identified, with gallic acid (up to 30.71 µg/mg), rutin (21.93 µg/mg) and hesperidin (14.98 µg/mg) among the most abundant. The optimized ScCO_2_-150 extract in particular demonstrated a superior ability to concentrate key bioactive compounds such as gallic acid, rutin and hesperidin, establishing a strong link between extraction parameters and phytochemical yield.

The ScCO_2_-150 extract demonstrated the highest antimicrobial activity, particularly against *S. aureus B. subtilis* and *P. aeruginosa*, with inhibition zones reaching up to 21.3 mm (*B. subtilis*) and MIC values ranging from 125 to 250 µg/mL. For the first time the antibiofilm potential of *R. cordatum* extracts was evaluated, revealing that UAE-E-4h and Sc-60 were the most effective, with BIC_90_ values of 1346.58 µg/mL and 1339.15 µg/mL, respectively. These findings underscore the importance of the extraction strategy in selectively isolating bioactive compounds with both antimicrobial and antibiofilm activities.

The findings of this study expand the current understanding of the biological potential of *R. cordatum* aerial parts which have been under-explored in the literature. Utilizing leaves instead of roots promotes the sustainable use of plant resources without compromising natural populations. Collectively, the results highlight the high potential of *R. cordatum* as a source of natural antioxidant and antimicrobial agents, and emphasize the advantages of supercritical CO_2_ extraction as an efficient and environmentally friendly method for obtaining bioactive compounds.

## Figures and Tables

**Table 1:** Quantitative LC-MS/MS profile of phytochemicals in *Rheum cordatum* Losinsk. leaf extracts.

No	Class	Analytes	UAE-M-1h	UAE-M-4h	UAE-E-1h	UAE-E-4h	Sc-60	Sc-80	ScCO_2_-100	ScCO_2_-150
1	Organic acid	Quinic acid	513	3310	257	1621	2575	3676	60	289
		Fumaric aid	N.D.	224	N.D.	210	240	N.D.	N.D.	N.D.
		Aconitic acid	N.D.	N.D.	N.D.	912	21	3463	N.D.	N.D.
2	Phenolic acid	Gallic acid	7619	17,977	12,759	30,705	17,398	4094	11,250	17,304
		Protocatechuic acid	102	282	207	3539	282	3461	307	430
		4-OH Benzoic acid	N.D.	N.D.	N.D.	334	N.D.	282	N.D.	N.D.
		Caffeic acid	28	53	38	612	57	60	102	131
		Syringic acid	N.D.	N.D.	N.D.	298	N.D.	N.D.	129	N.D.
		p-Coumaric acid	20	64	43	179	50	35	122	130
		Ferulic acid	N.D.	N.D.	N.D.	126	N.D.	N.D.	N.D.	66
		Salicylic acid	N.D.	N.D.	N.D.	65	N.D.	29	N.D.	N.D.
3	Phenolic aldehyde	Protocatechuic aldehyde	N.D.	10	13	40	8	49	N.D.	12
4	Tannin	Tannic acid	388	638	93	93	427	58	52	59
5	Flavanols	Epigallocatechin gallate	846	1378	1670	N.D.	1899	N.D.	N.D.	2293
		Epicatechin gallate	2689	7546	4584	N.D.	6846	N.D.	195	5465
6	Flavonol glycoside	Rutin	8476	19,095	13,691	9317	20,471	1530	2990	21,932
		isoquercitrin	582	994	1089	638	974	121	333	1135
7	Flavanone glycoside	Hesperidin	5877	13,635	10,265	6724	13,779	1176	1957	14,975
8	Flavonoid glycoside	Astragalin	184	125	309	87	140	N.D.	93	245
		Nicotiflorin	1667	1693	2876	1003	1834	134	674	2622
9	Flavonol	Quercetin	117	303	290	150	223	40	278	425
		Kaempferol	N.D.	9	13	N.D.	8	N.D.	21	22
10	Flavonone	Naringenin	5	6	8	7	6	2	14	26
11	Flavone	Luteolin	N.D.	5	5	8	4	N.D.	5	8
		Apigenin	N.D.	N.D.	N.D.	7	N.D.	N.D.	N.D.	4
		Chrysin	N.D.	N.D.	N.D.	N.D.	N.D.	N.D.	17	42
		Acacetin	N.D.	N.D.	N.D.	219	N.D.	N.D.	3	4
12	Biflavonoid	Amentoflavone	N.D.	N.D.	N.D.	7	N.D.	N.D.	N.D.	N.D.
13	Internal standards	Ferulic acid-D3-IS	N.A.	N.A.	N.A.	N.A.	N.A.	N.A.	N.A.	N.A.
		Rutin-D3-IS	N.A.	N.A.	N.A.	N.A.	N.A.	N.A.	N.A.	N.A.
		Quercetin-D3-IS	N.A.	N.A.	N.A.	N.A.	N.A.	N.A.	N.A.	N.A.

All concentrations are given in µg/g. N.D.—not detected; compound concentration was below the LOD determined for each analyte (see Supplementary Table S1). N.A.—not applicable; the compound was used as an internal standard.

**Table 2:** Radical scavenging (IC_50_ mg/mL) and total phenolic substance amounts (mg GAE/g).

Samples	DPPH (IC_50_ mg/mL)	R^2^	ABTS (IC_50_ mg/mL)	R^2^	Total Phenolic (mg GAE/g)
BHA *	0.0023	0.994	0.0021 ± 0.001	0.981	-
BHT *	0.0038	0.961	0.0034 ± 0.001	0.993	-
Trolox *	0.0076	0.991	0.0041 ± 0.002	0.998	-
UAE-M-1h	0.0872 ± 0.003	0.99	0.0684 ± 0.0021	0.99	78 ± 3
UAE-M-4h	0.0472 ± 0.003	0.982	0.0612 ± 0.0023	0.981	100 ± 2
UAE-E-1h	0.0772 ± 0.003	0.995	0.0673 ± 0.0025	0.992	82 ± 2
UAE-E-4h	0.0570 ± 0.004	0.989	0.0645 ± 0.003	0.985	95 ± 2
Sc-60	0.0379 ± 0.003	0.993	0.0521 ± 0.0021	0.992	125 ± 3
Sc-80	0.1042 ± 0.001	0.982	0.0792 ± 0.0029	0.988	52 ± 4
ScCO_2_-100	0.0972 ± 0.003	0.983	0.0682 ± 0.0024	0.990	68 ± 3
ScCO_2_-150	0.0132 ± 0.002	0.99	0.0462 ± 0.0033	0.985	140 ± 5

* Standard antioxidants used as positive controls.

**Table 3:** Inhibition zone diameters of extracts tested against pathogenic strains.

	UAE-M-1h	UAE-M-4h	UAE-E- 1h	UAE-E-4h	Sc-60	Sc-80	ScCO_2_-100	ScCO_2_-150
*E. feacalis*	10.5	0.0	0.0	0.0	0.0	0.0	14.9	13.9
*B. spizizenii*	9	0.0	0.0	0.0	9.7	0.0	14.6	21.3
*K. pneumonia*	8.4	0.0	0.0	0.0	0.0	0.0	13.1	16.8
*P. aeruginosa*	11.6	10.9	0.0	9	0.0	0.0	17.4	17.8
*E. coli*	8.8	0.0	0.0	9.6	11.4	10.3	0.0	0.0
*S. aureus*	9.3	0.0	0.0	13.5	0.0	0.0	15.0	17.1
*C. albicans*	0.0	0.0	0.0	0.0	0.0	0.0	0.0	0.0
*C. tropicalis*	8.8	0.0	0.0	0.0	0.0	0.0	0.0	18.1

“0.0” indicates that the extract was tested but no inhibition zone was observed under the experimental conditions.

**Table 4:** Minimum inhibitory concentrations (MICs) and minimum bactericidal concentrations (MBCs) of *R. cordatum* leaf extracts.

	*E. feacalis*		*B. subtilis*		*K. pneumoniae*		*P. aeruginosa*		*S. aureus*		*C. tropicalis*		*E.coli*	
	**MİC**	**MBC**	**MİC**	**MBC**	**MİC**	**MBC**	**MİC**	**MBC**	**MİC**	**MBC**	**MİC**	**MBC**	**MİC**	**MBC**
UAE-M-1h	125	1000	1000	>1000	500	>1000	250	>1000	1000	>1000	250	1000	1000	>1000
UAE-M-4h	*	*	*	*	*	*	250	1000	*	*	*	*	*	*
UAE- E-1h	*	*	*	*	*	*	*	*	*	*	*	*	*	*
UAE-E-4h	*	*	*	*	*	*	500	>1000	500	>1000	*	*	500	>1000
Sc-60	*	*	250	>1000	*	*	*	*	*	*	*	*	500	>1000
Sc-80	*	*	*	*	*	*	*	*	*	*	*	*	500	>1000
ScCO_2_-100	500	1000	500	1000	500	>1000	125	500	500	1000	*	*	*	*
ScCO_2_-150	250	1000	125	500	250	1000	125	500	125	500	250	1000	*	*

*: not tested. Groups showing no MBC effect at the highest concentration tested are indicated by a > sign.

**Table 5:** Biofilm inhibition concentration (BIC_90_ values µg/mL).

Extraction	*E. feacalis*	*B. subtilis*	*K. pneumoniae*	*P. aeruginosa*
UAE-M-1h	166,107	551,236	176,198	305,814
UAE-M-4h	n.d.	n.d.	n.d.	137,312
UAE-E-4h	n.d.	n.d.	n.d.	134,658
Sc-60	n.d.	133,915	n.d.	n.d.
ScCO_2_-100	220,864	188,262	263,659	165,168
ScCO_2_-150	301,420	285,871	262,642	226,610

n.d.: BIC_90_ could not be determined due to a lack of biofilm inhibition by the extract.

## Data Availability

Data are contained within the article and Supplementary Materials.

## Supplementary Materials

The following supporting information can be downloaded at https://www.mdpi.com/article/10.3390/plants14152314/s1: Figure S1: LC-MS/MS chromatogram of the standardized LC-MS/MS method; Table S1: Analytical method validation parameters of the LC-MS/MS method for the quantification of 53 phytochemicals; Figure S2: LC-MS/MS chromatograms of *Rheum cordatum* Losinsk leaf extracts obtained by various extraction methods (A. UAE-M-1h; B. UAE-M-4h; C. UAE-E-1h; D. UAE-E-4h; E. Sc-60; F. Sc-80; G. ScCO_2_-100; H. ScCO_2_-150).
