# Supplementary material for: Bioactive Potential of Rheum cordatum Losinsk. Leaf Extracts: Phytochemical Insights from Supercritical CO2, Subcritical Ethanol and Ultrasound-Assisted Extractions
Source: Plants (Basel). 2025 Jul 26;14(15):2314. doi: 10.3390/plants14152314 (PMC12349647; doi:10.3390/plants14152314)
Supplement: Supplementary file 1 [file plants-14-02314-s001.zip › plants-3753715-supplementary.pdf]

Bioactive Potential of *Rheum cordatum* Losinsk. Leaf Extracts: Phytochemical Insights from Supercritical CO_2, Subcritical Ethanol and Ultrasound-Assisted Extractions

Madina Amangeldinova [1,2], Mehmet Ersatır [3], Pınar Küce Cevik [4], Mustafa Abdullah Yilmaz [5,6], Oguz Cakır [5,7], Nataliya Kudrina [1,2,*], Aizhan Mussayeva [2], Timur Kulmanov [2], Nina Terletskaya [1,2] and Metin Yildirim [8,*]

[1] Faculty of Biology and Biotechnology, Al-Farabi Kazakh National University, Almaty 050040, Kazakhstan; madu.ma@mail.ru (M.A.); teni02@mail.ru (N.T.)
[2] Institute of Genetic and Physiology, Al-Farabi 93, Almaty 050040, Kazakhstan; aimus_@mail.ru (A.M.); kulmanovlux@mail.ru (T.K.)
[3] Department of Chemistry, Faculty of Art and Science, Cukurova University, Adana 01330, Türkiye;
mehmetersatir8@gmail.com
[4] Department of Molecular Biology and Genetic, Faculty of Science and Arts, Harran University, Sanliurfa 63290, Turkey; pinarkcvk@harran.edu.tr
[5] Science and Technology Research and Application Center, Dicle University, Diyarbakir 21280, Türkiye;
mustafaabdullahyilmaz@gmail.com
[6] Department of Analytical Chemistry, Faculty of Pharmacy, Dicle University, Diyarbakir 21280, Türkiye
[7] Department of Nutrition and Dietetics, Atatürk Faculty of Health Sciences, Dicle University
Diyarbakir 21280, Türkiye; ocakir44@gmail.com
[8] Department of Biochemistry, Faculty of Pharmacy, Harran University, Sanliurfa 63290, Türkiye

* Correspondence: kudrina_nat@mail.ru (N.K.); metinyildirim4@gmail.com (M.Y.);
Tel.:+7-705-181-14-40 (N.K.);+90-537-527-44-43 (M.Y.)

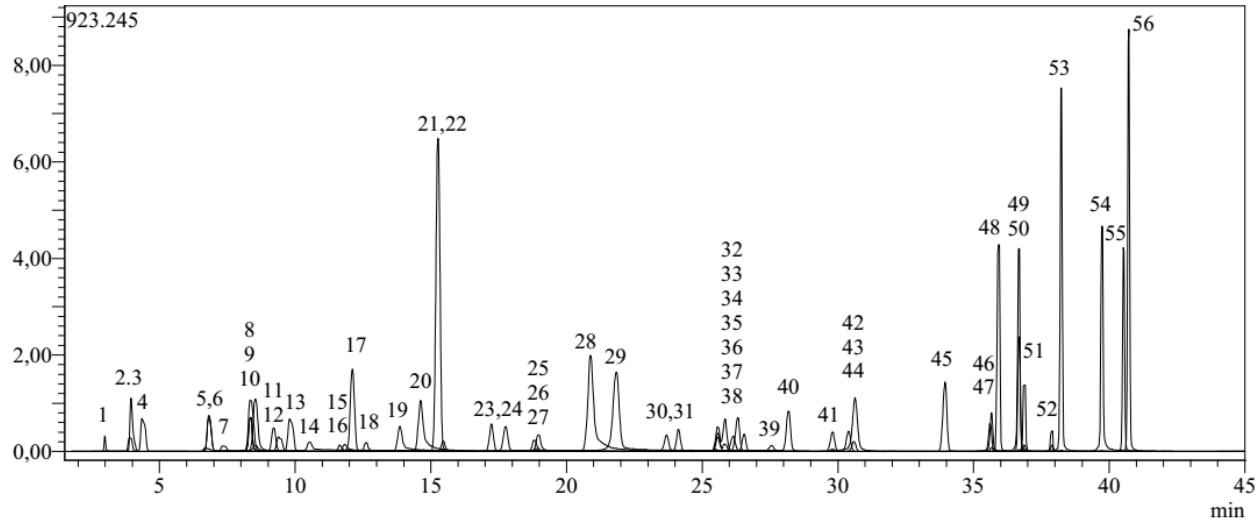

Figure S1. LC-MS/MS chromatogram of the standardized LC-MS/MS method

Mass spectrometer and LC-MS/MS method

A Shimadzu-Nexera model ultrahigh performance liquid chromatography (UHPLC) coupled with a tandem mass spectrometer was used to accomplish quantitative evaluation of 53 phytochemicals. The reversed-phase UHPLC was equipped with an autosampler (SIL-30AC model), a column oven (CTO-10ASvp model), binary pumps (LC-30AD model), and a degasser (DGU- 20A3R model). The chromatographic conditions were optimized to achieve optimum separation for 53 phytochemicals and overcome the suppression effects. Different coluımns such as Agilent Poroshell 120 EC-C18 model (150 mm×2.1 mm, 2.7 µm) and RP-C18 Inertsil ODS-4 (100 mm×2,1 mm, 2µm), different mobile phases (B) such as acetonitrile and methanol, different mobile phase additives such as ammonium formate, formic acid, ammonium acetate, and acetic acid, different column temperatures such as 25°C, 30°C, 35°C and 40°C were tried and applied until the optimum conditions were achieved. Consequently, the chromatographic separation was performed on a reversed-phase Agilent Poroshell 120 EC-C18 model (150 mm×2.1 mm, 2.7 µm) analytical column. The column temperature was set to 40°C. The elution gradient was composed of eluent A (water+5 mM ammonium formate+0.1% formic acid) and eluent B (methanol+5 mM ammonium formate+0.1% formic acid). The following gradient elution profile was used: 20-100% B (0-25 min), 100% B (25-35 min), 20% B (35-45 min). Furthermore, the solvent flow rate and injection volume were settled as 0.5 mL/min and 5 µL, respectively.

The mass spectrometric detection was carried out using a Shimadzu LCMS-8040 model tandem mass spectrometer equipped with an electrospray ionization (ESI) source operating in both negative and positive ionization modes. LC-ESI-MS/MS data were acquired and processed by LabSolutions software (Shimadzu). The MRM (multiple reaction monitoring) mode was used for the quantification of the phytochemicals. The MRM method was optimized to selectively detect and quantify phytochemical compounds based on the screening of specified precursor phytochemical-to-fragment ion transitions. The collision energies (CE) were optimized in order to generate optimal photochemical fragmentation and maximal transmission of the desired product ions. The MS operating conditions were applied as: drying gas (N2) flow,

15 L/min; nebulizing gas (N_2) flow, 3 L/min; DL temperature, 250°C; heat block temperature, 400°C, and interface temperature, 350°C.

Table S1. Analytical method validation parameters that belong to the LC-MS/MS method

No	Analytes	RT[a]	M.I. (m/z)[b]	F.I. (m/z)[c]	Ion. mode	Equation	r^{2d}	*RSD*%[e]		Linearity Range (mg/L)	*LOD*/*LOQ* (µg/L)[f]	Recovery (%)		*U*[g]	Gr. No[i]
								Interday	Intraday			Interday	Intraday		
1	Quinic acid	3.0	190.8	93.0	Neg	*y*=-0.0129989+2.97989×	0.996	0.69	0.51	0.1-5	25.7/33.3	1.0011	1.0083	0.0372	1
2	Fumaric aid	3.9	115.2	40.9	Neg	*y*=-0.0817862+1.03467×	0.995	1.05	1.02	1-50	135.7/167.9	0.9963	1.0016	0.0091	1
3	Aconitic acid	4.0	172.8	129.0	Neg	*y*=-0.7014530+32.9994×	0.971	2.07	0.93	0.1-5	16.4/31.4	0.9968	1.0068	0.0247	1
4	Gallic acid	4.4	168.8	79.0	Neg	*y*=0.0547697+20.8152×	0.999	1.60	0.81	0.1-5	13.2/17.0	1.0010	0.9947	0.0112	1
5	Epigallocatechin	6.7	304.8	219.0	Neg	*y*=-0.00494986+0.0483704×	0.998	1.22	0.73	1-50	237.5/265.9	0.9969	1.0040	0.0184	3
6	Protocatechuic acid	6.8	152.8	108.0	Neg	*y*=0.211373+12.8622×	0.957	1.43	0.76	0.1-5	21.9/38.6	0.9972	1.0055	0.0350	1
7	Catechin	7.4	288.8	203.1	Neg	*y*=-0.00370053+0.431369×	0.999	2.14	1.08	0.2-10	55.0/78.0	1.0024	1.0045	0.0221	3
8	Gentisic acid	8.3	152.8	109.0	Neg	*y*=-0.0238983+12.1494×	0.997	1.81	1.22	0.1-5	18.5/28.2	0.9963	1.0077	0.0167	1
9	Chlorogenic acid	8.4	353.0	85.0	Neg	*y*=0.289983+36.3926×	0.995	2.15	1.52	0.1-5	13.1/17.6	1.0000	1.0023	0.0213	1
10	Protocatechuic aldehyde	8.5	137.2	92.0	Neg	*y*=0.257085+25.4657×	0.996	2.08	0.57	0.1-5	15.4/22.2	1.0002	0.9988	0.0396	1
11	Tannic acid	9.2	182.8	78.0	Neg	*y*=0.0126307+26.9263×	0.999	2.40	1.16	0.05-2.5	15.3/22.7	0.9970	0.9950	0.0190	1
12	Epigallocatechin gallate	9.4	457.0	305.1	Neg	*y*=-0.0380744+1.61233×	0.999	1.30	0.63	0.2-10	61.0/86.0	0.9981	1.0079	0.0147	3
13	1,5-dicaffeoylquinic acid	9.8	515.0	191.0	Neg	*y*=-0.0164044+16.6535×	0.999	2.42	1.48	0.1-5	5.8/9.4	0.9983	0.9997	0.0306	1
14	4-OH Benzoic acid	10.5	137,2	65.0	Neg	*y*=-0.0240747+5.06492×	0.999	1.24	0.97	0.2-10	68.4/88.1	1.0032	1.0068	0.0237	1
15	Epicatechin	11.6	289.0	203.0	Neg	*y*=-0.0172078+0.0833424×	0.996	1.47	0.62	1-50	139.6/161.6	1.0013	1.0012	0.0221	3
16	Vanilic acid	11.8	166.8	108.0	Neg	*y*=-0.0480183+0.779564×	0.999	1.92	0.76	1-50	141.9/164.9	1.0022	0.9998	0.0145	1
17	Caffeic acid	12.1	179.0	134.0	Neg	*y*=0.120319+95.4610×	0.999	1.11	1.25	0.05-2.5	7.7/9.5	1.0015	1.0042	0.0152	1
18	Syringic acid	12.6	196.8	166.9	Neg	*y*=-0.0458599+0.663948×	0.998	1.18	1.09	1-50	82.3/104.5	1.0006	1.0072	0.0129	1
19	Vanillin	13.9	153.1	125.0	Poz	*y*=0.00185898+20.7382×	0.996	1.10	0.85	0.1-5	24.5/30.4	1.0009	0.9967	0.0122	1
20	Syringic aldehyde	14.6	181.0	151.1	Neg	*y*=-0.0128684+7.90153×	0.999	2.51	0.77	0.4-20	19.7/28.0	1.0001	0.9964	0.0215	1
21	Daidzin	15.2	417.1	199.0	Poz	*y*=9.45747+152.338×	0.996	2.25	1.32	0.05-2.5	7.0/9.5	0.9955	1.0017	0.0202	2
22	Epicatechin gallate	15.5	441.0	289.0	Neg	*y*=-0.0142216+1.06768×	0.997	1.63	1.28	0.1-5	19.5/28.5	0.9984	0.9946	0.0229	3
23	Piceid	17.2	391.0	135/106.9	Poz	*y*=0.00772525+25.4181×	0.999	1.94	1.16	0.05-2.5	13.8/17.8	1.0042	0.9979	0.0199	1
24	*p*-Coumaric acid	17.8	163.0	93.0	Neg	*y*=0.0249034+18.5180×	0.999	1.92	1.43	0.1-5	25.9/34.9	1.0049	1.0001	0.0194	1
25	Ferulic acid-D3-IS[h]	18.8	196.2	152.1	Neg	N.A.	N.A.	N.A.	N.A.	N.A.	N.A.	N.A.	N.A.	0.0170	1
26	Ferulic acid	18.8	192.8	149.0	Neg	*y*=-0.0735254+1.34476×	0.999	1.44	0.53	1-50	11.8/15.6	0.9951	0.9976	0.0181	1
27	Sinapic acid	18.9	222.8	193.0	Neg	*y*=-0.0929932+0.836324×	0.999	1.45	0.52	0.2-10	65.2/82.3	1.0031	1.0037	0.0317	1
28	Coumarin	20.9	146.9	103.1	Poz	*y*=0.0633397+136.508×	0.999	2.11	1.54	0.05-2.5	214.2/247.3	0.9950	0.9958	0.0383	1

[a]R.T.: Retention time, [b]MI *(m/z):* Molecular ions of the standard analytes (m/z ratio), [c]FI *(m/z):* Fragment ions [d]r^2: Coefficient of determination, [e]*RSD*: Relative standard deviation, [f]*LOD*/*LOQ* (µg/L): Limit of detection/quantification, [g]*U* (%): percent relative uncertainty at 95% confidence level ($k = 2$), [h]IS: Internal standard, [i]Gr. No: Represents grouping of internal standards, these numbers indicate which IS stands for which phenolic compound.

No	Analytes	RT[a]	M.I. (m/z)[b]	F.I. (m/z)[c]	Ion. mode	Equation	r^{2d}	*RSD*%[e]		Linearity Range (mg/L)	*LOD*/*LOQ* (µg/L)[f]	Recovery (%)		*U*[g]	Gr. No
								Interday	Intraday			Interday	Intraday		
29	Salicylic acid	21.8	137.2	65.0	Neg	*y*=0.239287+153.659×	0.999	1.48	1.18	0.05-2.5	6.0/8.3	0.9950	0.9998	0.0158	1
30	Cynaroside	23.7	447.0	284.0	Neg	*y*=0.280246+6.13360×	0.997	1.56	1.12	0.05-2.5	12.1/16.0	1.0072	1.0002	0.0366	2
31	Miquelianin	24.1	477.0	150.9	Neg	*y*=-0.00991585+5.50334×	0.999	1.31	0.95	0.1-5	10.6/14.7	0.9934	0.9965	0.0220	2
32	Rutin-D3-IS[h]	25.5	612.2	304.1	Neg	N.A.	N.A.	N.A.	N.A.	N.A.	N.A.	N.A.	N.A.	N.A.	2
33	Rutin	25.6	608.9	301.0	Neg	*y*=-0.0771907+2.89868×	0.999	1.38	1.09	0.1-5	15.7/22.7	0.9977	1.0033	0.0247	2
34	isoquercitrin	25.6	463.0	271.0	Neg	*y*=-0.111120+4.10546×	0.998	2.13	0.78	0.1-5	8.7/13.5	1.0057	0.9963	0.0220	2
35	Hesperidin	25.8	611.2	449.0	Poz	*y*=0.139055+13.2785×	0.999	1.84	1.35	0.1-5	19.0/26.0	0.9967	1.0043	0.0335	2
36	*o*-Coumaric acid	26.1	162.8	93.0	Neg	*y*=0.00837193+11.2147×	0.999	2.11	1.46	0.1-5	31.8/40.4	1.0044	0.9986	0.0147	1
37	Genistin	26.3	431.0	239.0	Neg	*y*=1.65808+7.57459×	0.991	2.01	1.28	0.1-5	14.9/21.7	1.0062	1.0047	0.0083	2
38	Rosmarinic acid	26.6	359.0	197.0	Neg	*y*=-0.0117238+8.04377×	0.999	1.24	0.86	0.1-5	16.2/21.2	1.0056	1.0002	0.0130	1
39	Ellagic acid	27.6	301.0	284.0	Neg	*y*=0.00877034+0.663741×	0.999	1.57	1.23	0.4-20	56.9/71.0	1.0005	1.0048	0.0364	1
40	Cosmosiin	28.2	431.0	269.0	Neg	*y*=-0.708662+8.62498×	0.998	1.65	1.30	0.1-5	6.3/9.2	0.9940	0.9973	0.0083	2
41	Quercitrin	29.8	447.0	301.0	Neg	*y*=-0.00153274+3.20368×	0.999	2.24	1.16	0.1-5	4.8/6.4	0.9960	0.9978	0.0268	2
42	Astragalin	30.4	447.0	255.0	Neg	*y*=0.00825333+3.51189×	0.999	2.08	1.72	0.1-5	6.6/8.2	0.9968	0.9957	0.0114	2
43	Nicotiflorin	30.6	592.9	255.0/284.0	Neg	*y*=0.00499333+2.62351×	0.999	1.48	1.23	0.05-2.5	11.9/16.7	0.9954	1.0044	0.0108	2
44	Fisetin	30.6	285.0	163.0	Neg	*y*=0.0365705+8.09472×	0.999	1.75	1.19	0.1-5	10.1/12.7	0.9980	1.0042	0.0231	3
45	Daidzein	34.0	253.0	223.0	Neg	*y*=-0.0329252+6.23004×	0.999	2.18	1.73	0.1-5	9.8/11.6	0.9926	0.9963	0.0370	3
46	Quercetin-D3-IS[h]	35.6	304.0	275.9	Neg	N.A.	N.A.	N.A.	N.A.	N.A.	N.A.	N.A.	N.A.	N.A.	3
47	Quercetin	35.7	301.0	272.9	Neg	*y*=+0.00597342+3.39417×	0.999	1.89	1.38	0.1-5	15.5/19.0	0.9967	0.9971	0.0175	3
48	Naringenin	35.9	270.9	119.0	Neg	*y*=-0.00393403+14.6424×	0.999	2.34	1.69	0.1-5	2.6/3.9	1.0062	1.0020	0.0392	3
49	Hesperetin	36.7	301.0	136.0/286.0	Neg	*y*=+0.0442350+6.07160×	0.999	2.47	2.13	0.1-5	7.1/9.1	0.9998	0.9963	0.0321	3
50	Luteolin	36.7	284.8	151.0/175.0	Neg	*y*=-0.0541723+30.7422×	0.999	1.67	1.28	0.05-2.5	2.6/4.1	0.9952	1.0029	0.0313	3
51	Genistein	36.9	269.0	135.0	Neg	*y*=-0.00507501+12.1933×	0.999	1.48	1.19	0.05-2.5	3.7/5.3	1.0069	1.0012	0.0337	3
52	Kaempferol	37.9	285.0	239.0	Neg	*y*=-0.00459557+3.13754×	0.999	1.49	1.26	0.05-2.5	10.2/15.4	0.9992	0.9990	0.0212	3
53	Apigenin	38.2	268.8	151.0/149.0	Neg	*y*=0.119018+34.8730×	0.998	1.17	0.96	0.05-2.5	1.3/2.0	0.9985	1.0003	0.0178	3
54	Amentoflavone	39.7	537.0	417.0	Neg	*y*=0.727280+33.3658×	0.992	1.35	1.12	0.05-2.5	2.8/5.1	0.9991	1.0044	0.0340	3
55	Chrysin	40.5	252.8	145.0/119.0	Neg	*y*=-0.0777300+18.8873×	0.999	1.46	1.21	0.05-2.5	1.5/2.8	0.9922	1.0050	0.0323	3
56	Acacetin	40.7	283.0	239.0	Neg	*y*=-0.559818+163.062×	0.997	1.67	1.28	0.02-1	1.5/2.5	0.9949	1.0011	0.0363	3

[a]R.T.: Retention time, [b]MI (*m/z*): Molecular ions of the standard analytes (m/z ratio), [c]FI (*m/z*): Fragment ions [d]r^2: Coefficient of determination, [e]*RSD*: Relative standard deviation, [f]*LOD*/*LOQ* (µg/L): Limit of detection/quantification, [g]*U* (%): percent relative uncertainty at 95% confidence level ($k = 2$), [h]IS: Internal standard, [i]Gr. No: Represents grouping of internal standards, these numbers indicate which IS stands for which phenolic compound.

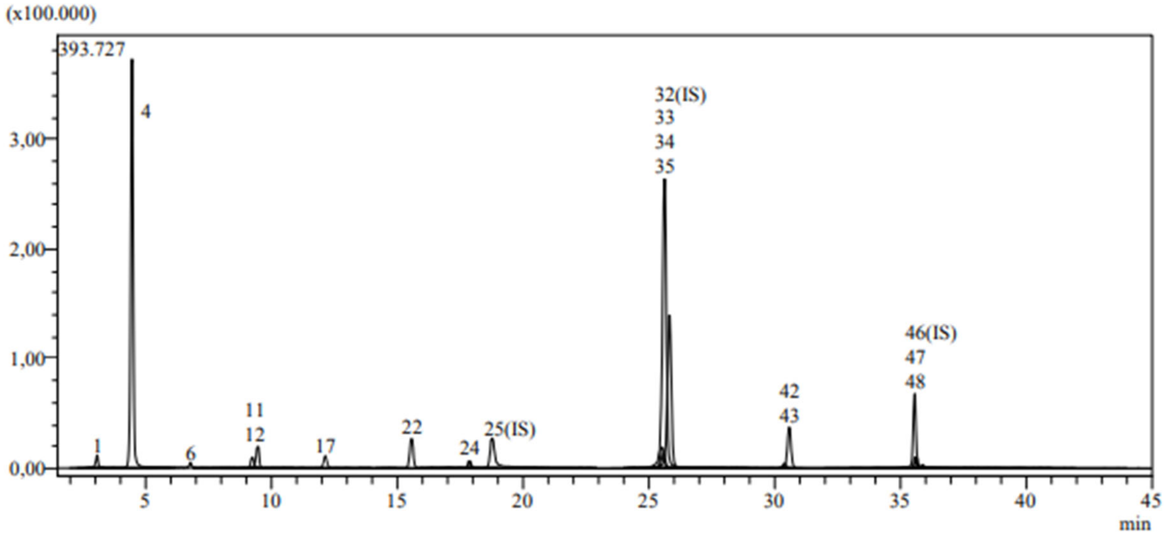
(x100.000)
393.727
3,00
2,00
1,00
0,00
1
4
6
11
12
17
22
24
25(IS)
32(IS)
33
34
35
42
43
46(IS)
47
48
5
10
15
20
25
30
35
40
45
min

(A)

1.lcd

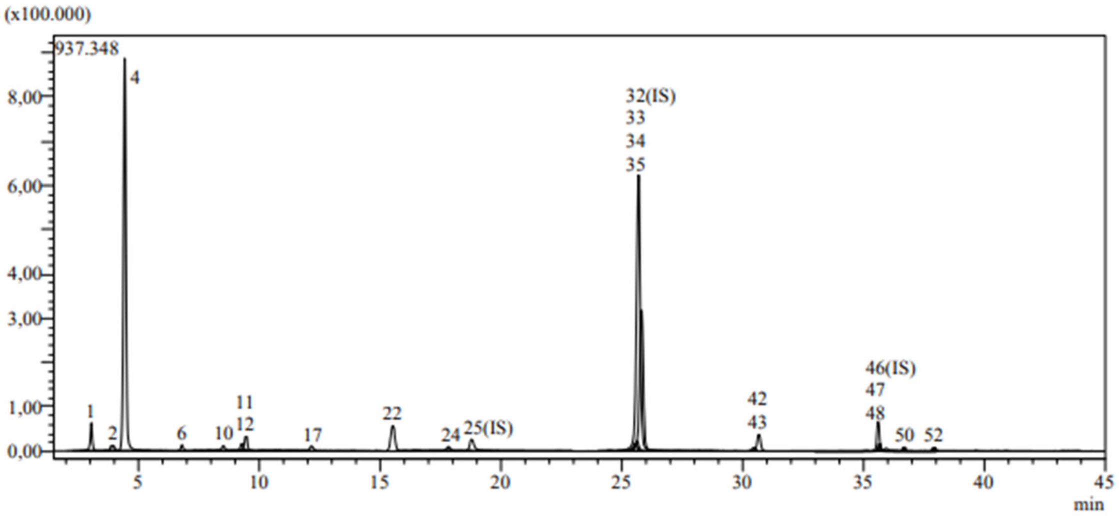
(x100.000)
937.348
8,00
6,00
4,00
3,00
1,00
0,00
1
2
4
6
10
11
12
17
22
24
25(IS)
32(IS)
33
34
35
42
43
46(IS)
47
48
50
52
5
10
15
20
25
30
35
40
45
min

(B)

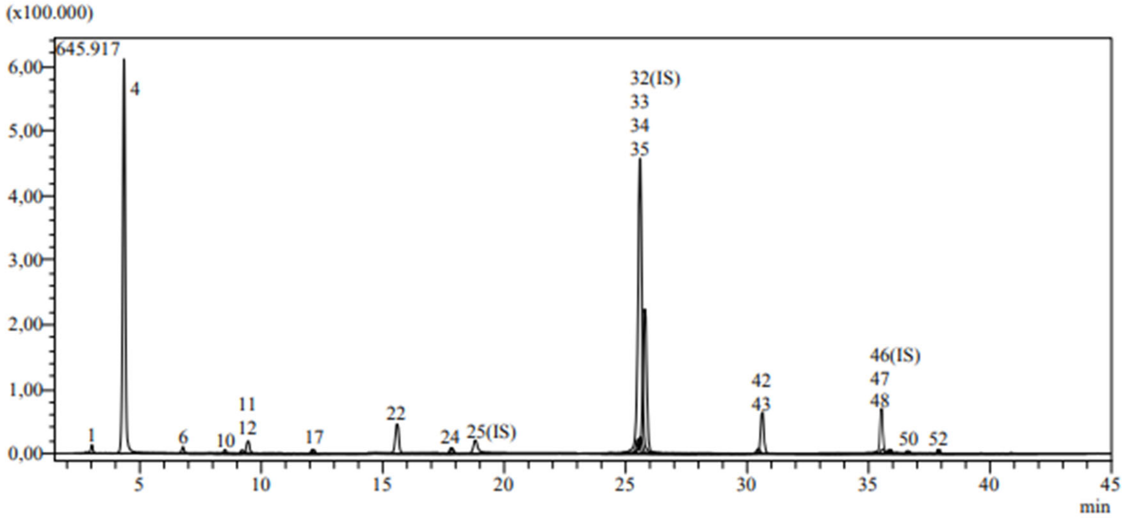
(x100.000)
645.917
6,00
5,00
4,00
3,00
2,00
1,00
0,00
1
4
6
10
11
12
17
22
24
25(IS)
32(IS)
33
34
35
42
43
46(IS)
47
48
50
52
5
10
15
20
25
30
35
40
45
min

(C)

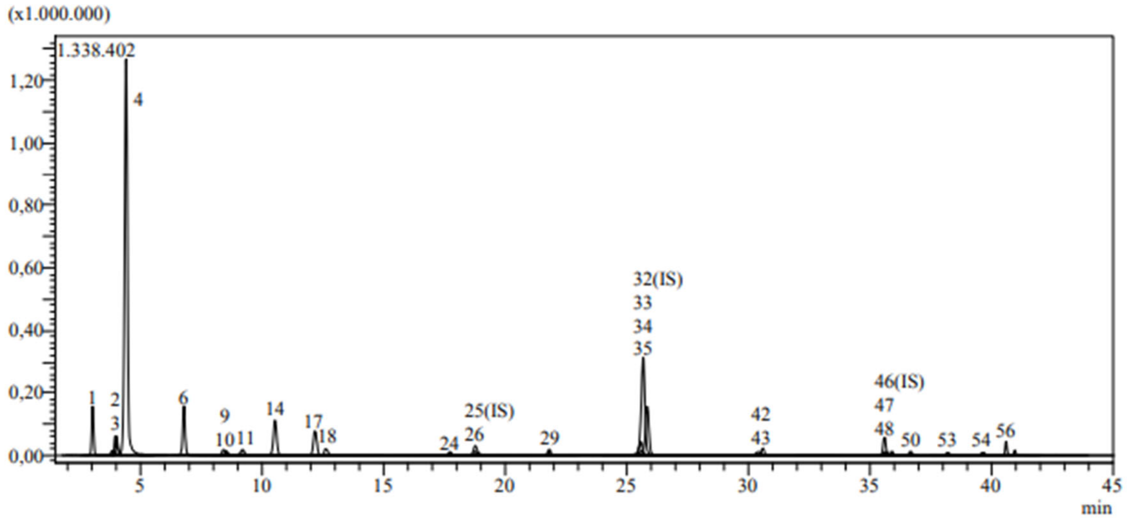

(x1.000.000)
1.338.402
1,20
1,00
0,80
0,60
0,40
0,20
0,00
1
2
3
4
6
9
10
11
14
17
18
24
25(IS)
26
29
32(IS)
33
34
35
42
43
46(IS)
47
48
50
53
54
56
5
10
15
20
25
30
35
40
45
min

(D)

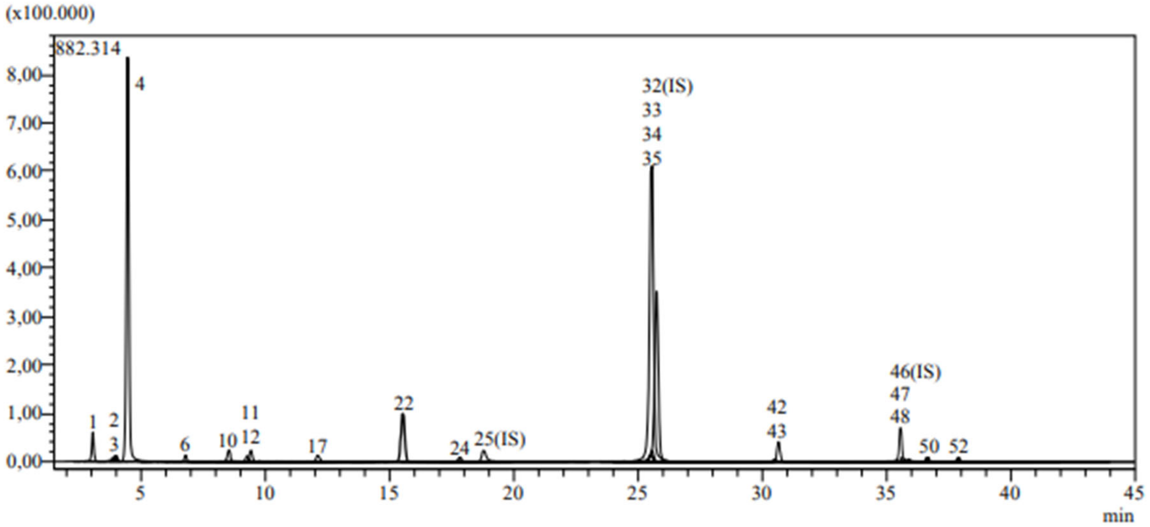

(x100.000)
882.314
8,00
7,00
6,00
5,00
4,00
3,00
2,00
1,00
0,00
1
2
3
4
6
10
11
12
17
22
24
25(IS)
32(IS)
33
34
35
42
43
46(IS)
47
48
50
52
5
10
15
20
25
30
35
40
45
min

(E)

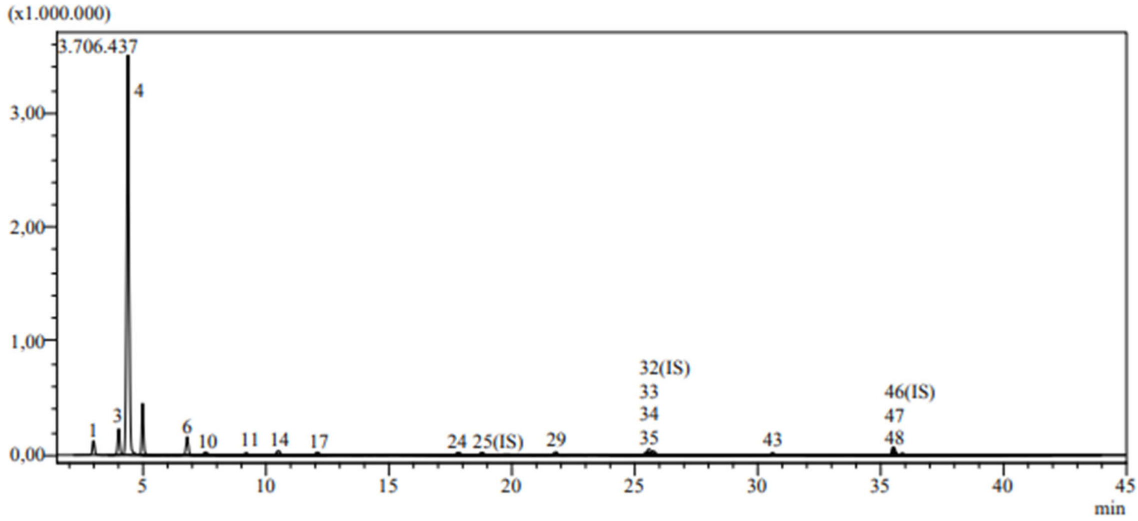
(x1.000.000)
3.706.437
3,00
2,00
1,00
0,00
4
1
3
6
10
11
14
17
24
25(IS)
29
32(IS)
33
34
35
43
46(IS)
47
48
5
10
15
20
25
30
35
40
45
min

(F)

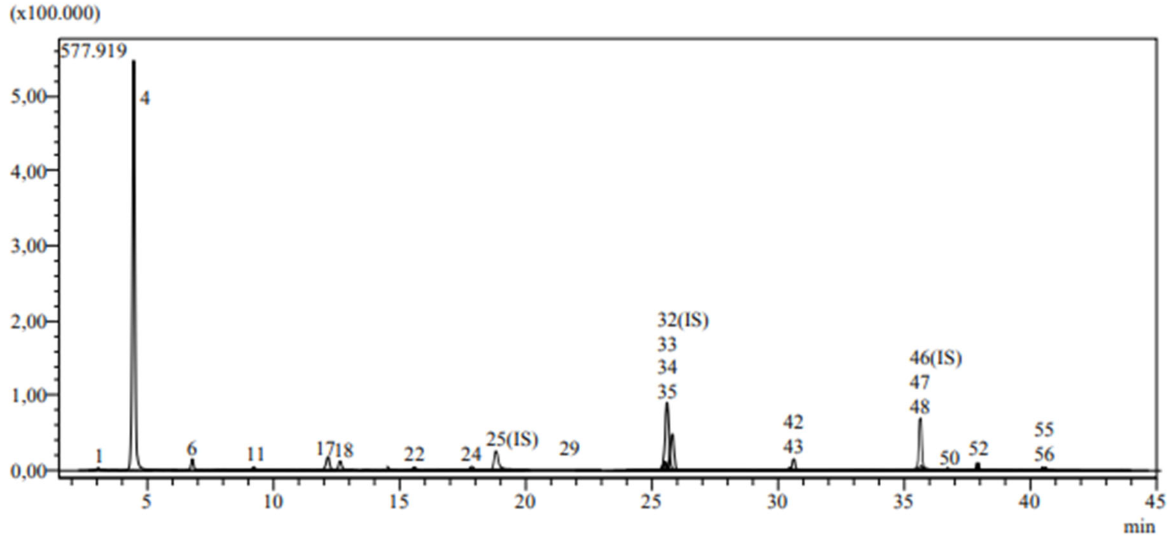
(x100.000)
577.919
5,00
4,00
3,00
2,00
1,00
0,00
4
1
6
11
17
18
22
24
25(IS)
29
32(IS)
33
34
35
42
43
46(IS)
47
48
50
52
55
56
5
10
15
20
25
30
35
40
45
min

(G)

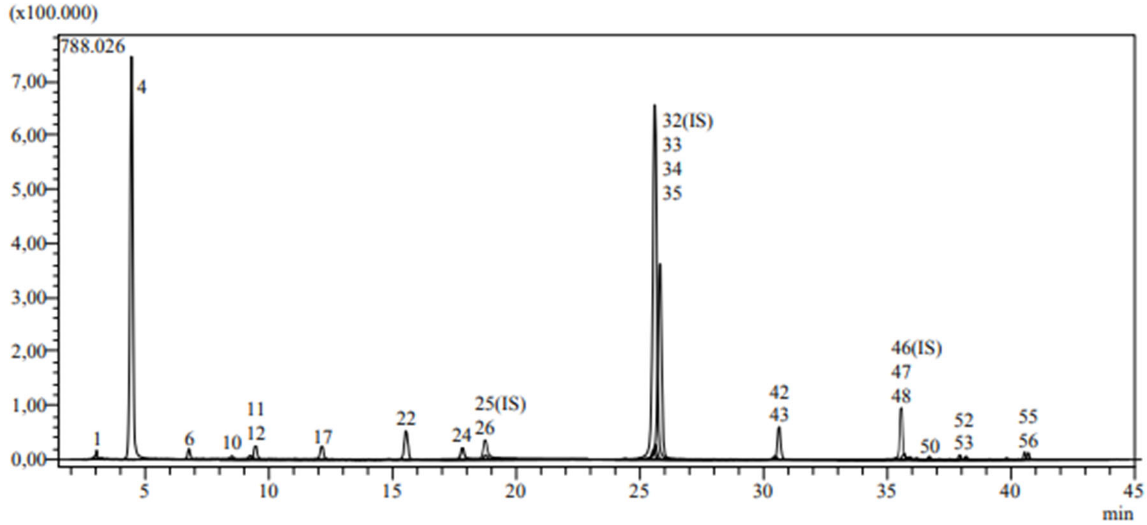
(x100.000)
788.026
7,00
6,00
5,00
4,00
3,00
2,00
1,00
0,00
4
1
6
10
11
12
17
22
24
25(IS)
26
32(IS)
33
34
35
42
43
46(IS)
47
48
50
52
53
55
56
5
10
15
20
25
30
35
40
45
min

(H)

Figure S2. LC/MS/MS chromatogram of the *Rheum cordatum* Losinsk leaves extracts

A. UAE-M-1h
B. UAE-M-4h
C. UAE-E-1h
D. UAE-E-4h
E. Sc-60
F. Sc-80
G. ScCO2-100
H. ScCO2-150
